# Prevalence of Sleep Duration on an Average School Night Among 4 Nationally Representative Successive Samples of American High School Students, 2007–2013

**DOI:** 10.5888/pcd11.140383

**Published:** 2014-12-11

**Authors:** Charles E. Basch, Corey H. Basch, Kelly V. Ruggles, Sonali Rajan

**Affiliations:** Author Affiliations: Corey H. Basch, William Paterson University, Wayne, New Jersey; Kelly V. Ruggles, New York University School of Medicine, New York, New York; Sonali Rajan, Columbia University, New York, New York.

## Abstract

Consistency, quality, and duration of sleep are important determinants of health. We describe sleep patterns among demographically defined subgroups from the Youth Risk Behavior Surveillance System reported in 4 successive biennial representative samples of American high school students (2007 to 2013). Across the 4 waves of data collection, 6.2% to 7.7% of females and 8.0% to 9.4% of males reported obtaining 9 or more hours of sleep. Insufficient duration of sleep is pervasive among American high school students. Despite substantive public health implications, intervention research on this topic has received little attention.

## Objective

Recent research provides evidence for conventional wisdom that consistency, quality, and duration of sleep are important determinants of health. Among adults, insufficient quality or duration of sleep is associated with obesity and diabetes (1), cardiovascular disease (1,2), and all-cause mortality (3). In early childhood (4) and among adolescents (5,6) and adults (7), sleep affects cognition. Sleep is essential for the health of the human brain (5). Despite the importance of sleep for cognition, development, and well-being, we did not find any studies describing duration of sleep among demographic subgroups of high school students in the United States during the past decade. We, therefore, used data from 4 successive biennial samples from the Youth Risk Behavior Surveillance System (YRBSS), representative of American high school students, to describe sleep patterns among demographically defined subgroups.

## Methods

We conducted secondary analyses of YRBSS data, weighted to align with national population proportions and collected in 2007, 2009, 2011, and 2013. We focused on the sleep duration item; added in 2007, the question was identical over time, “On an average school night, how many hours of sleep do you get?” We summarized the percentages of respondents by sex, grade, and race/ethnicity as 5 hours or less, 6 hours, 7 hours, 8 hours, or 9 hours or more. Data on participants with missing responses (<5%) were excluded. The number of respondents (and overall response rate [school response rate × student response rate]) for each year was 12,154 (68%) in 2007; 14,782 (71%) in 2009; 12,198 (71%) in 2011; and 13,584 (68%) in 2013. We assessed significant differences between percentages by using 95% confidence intervals (CIs). (CIs are available upon request from the authors.) Data were cleaned in Perl version 5.12.3 (www.perl.org) and analyzed using Matlab (MathWorks). This study was approved by the institutional review boards at Teachers College, Columbia University; William Paterson University; and New York University Langone Medical Center.

## Results

Across the 4 waves of data collection, 6.2% to 7.7% of females and 8.0% to 9.4% of males reported obtaining 9 or more hours of sleep. For all females across the 4 waves, we found a consistent pattern in which the percentage who reported obtaining 9 or more hours of sleep decreased as grade level increased (Table 1). This pattern was generally consistent when the data were disaggregated for white females, Hispanic females, and black females (Table 1). We found the same general pattern for males (Table 2). Without exception, compared with 9th graders, a significantly lower percentage of 11th and 12th graders reported obtaining 9 or more hours of sleep and, except in 2009 and 2013, a significantly higher percentage of 11th and 12th graders reported obtaining 5 or fewer hours of sleep.

**Table 1:** Hours of Sleep on an Average School Night Reported by a Representative Sample of Female High School Students in the United States, Youth Risk Behavior Surveillance System, 2007–2013^a^

Group	2007					2009					2011					2013				
	≤5 h	6 h	7 h	8 h	≥9 h	≤5 h	6 h	7 h	8 h	≥9 h	≤5 h	6 h	7 h	8 h	≥9 h	≤5 h	6 h	7 h	8 h	≥9 h
**Overall**	17.0	25.2	29.1	22.5	6.3	17.5	23.6	30.7	22.0	6.2	18.8	23.5	28.5	22.2	6.9	20.9	22.7	27.6	21.2	7.7
**Race/ethnicity**																				
White	15.0	25.6	31.9	22.5	5.0	15.2	24.6	33.6	22.0	4.6	15.8	23.6	30.4	23.6	6.5	18.7	22.6	29.3	23.1	6.3
Black	22.0	26.6	21.7	21.8	7.9	23.6	20.9	22.7	22.7	10.1	26.3	22.0	23.8	19.9	8.1	24.6	24.5	23.3	16.3	11.4
Hispanic	18.3	21.3	26.9	23.9	9.5	16.2	21.9	30.0	23.2	8.8	21.0	23.7	27.6	20.0	7.7	21.3	21.8	26.7	21.5	8.7
Other	19.9	26.4	25.9	20.7	7.1	24.8	26.1	27.4	18.0	3.7	24.0	24.5	25.0	20.7	5.8	27.4	24.3	22.8	17.6	7.8
**Grade**																				
9th	12.7	20.2	27.7	28.2	11.2	15.4	18.4	30.0	26.1	10.0	15.2	22.0	26.0	27.2	9.6	19.4	19.8	26.1	24.2	10.6
10th	16.6	24.2	30.0	23.5	5.7	17.7	21.6	32.0	23.0	5.7	18.0	22.1	29.2	23.4	7.3	19.7	20.1	30.3	21.2	8.7
11th	19.2	28.3	29.8	18.7	4.0	19.3	24.3	31.0	20.8	4.7	20.0	26.4	29.0	18.9	5.7	21.8	25.1	25.5	21.2	6.4
12th	20.1	29.2	28.9	18.5	3.4	17.7	31.1	29.8	17.6	3.7	22.7	23.9	30.6	18.5	4.3	22.5	26.5	28.7	17.9	4.5
**White, by grade**																				
9th	10.3	20.3	30.9	29.7	8.9	14.8	17.9	32.9	26.0	8.3	12.9	22.3	26.2	29.8	8.7	17.6	21.4	25.4	26.4	9.1
10th	17.7	22.4	33.1	22.2	4.6	15.0	23.0	34.8	23.5	3.9	16.2	21.2	30.5	24.8	7.3	17.5	19.3	32.7	23.1	7.4
11th	15.7	30.5	32.5	18.3	3.0	16.1	25.4	34.1	20.6	3.8	14.7	27.2	33.1	19.4	5.5	21.1	24.2	28.3	21.5	4.9
12th	16.8	29.8	31.2	18.8	3.3	15.2	32.1	31.9	18.1	2.6	19.6	24.0	32.4	19.7	4.4	18.5	25.5	31.0	21.3	3.6
**Black, by grade**																				
9th	17.1	22.7	20.2	27.2	12.8	18.0	16.4	22.7	28.7	14.2	20.3	18.2	22.2	25.9	13.3	22.0	18.4	25.0	17.9	16.6
10th	15.0	29.0	22.3	26.9	6.8	27.0	18.0	22.8	22.5	9.7	20.1	25.4	26.5	20.4	7.6	23.2	23.2	24.1	17.3	12.2
11th	27.1	29.0	22.1	16.0	5.8	25.9	20.4	23.7	22.1	7.9	33.1	20.7	21.8	17.4	7.1	24.8	28.1	20.0	17.9	9.1
12th	32.3	26.6	22.6	14.0	4.5	24.9	31.2	21.8	15.2	6.9	33.3	23.6	24.4	15.2	3.6	29.3	29.5	24.0	11.1	6.1
**Hispanic, by grade**																				
9th	16.4	18.5	24.9	23.8	16.3	14.1	19.2	28.3	26.3	12.1	19.1	22.1	26.3	21.7	10.8	17.3	19.3	29.3	24.3	9.8
10th	14.1	22.2	27.3	27.9	8.5	15.1	21.2	31.5	23.1	9.1	19.4	20.8	30.7	22.5	6.7	22.0	18.5	28.9	19.5	11.1
11th	19.3	20.1	30.0	23.3	7.3	17.7	24.2	28.9	22.4	6.8	23.4	28.0	23.1	18.7	6.8	20.3	26.3	20.7	25.3	7.4
12th	25.4	26.1	25.8	19.7	3.0	18.4	24.2	31.7	20.0	5.6	21.9	25.7	31.8	16.3	4.3	26.1	24.3	27.9	16.4	5.3
**Other race/ethnicity, by grade**																				
9th	14.9	14.6	29.3	25.6	15.6	16.6	25.1	31.0	21.7	5.6	14.3	24.2	28.7	25.3	7.6	29.6	14.4	23.5	20.2	12.3
10th	16.0	34.3	25.5	17.1	7.1	26.5	19.7	29.4	20.0	4.4	20.4	29.1	24.1	18.0	8.5	24.3	24.8	24.0	19.9	7.1
11th	30.9	25.0	23.3	18.3	2.6	34.1	24.2	24.7	15.6	1.5	32.0	23.9	21.4	19.8	2.9	25.7	28.2	21.9	18.9	5.2
12th	15.8	33.0	26.0	22.6	2.7	23.0	37.9	23.3	13.2	2.6	32.6	20.6	25.1	18.1	3.6	30.2	33.3	21.4	9.6	5.4

a All values are percentages.

**Table 2:** Hours of Sleep on an Average School Night Reported by a Representative Sample of Male High School Students in the United States, Youth Risk Behavior Surveillance System, 2007–2013^a^

Group	2007					2009					2011					2013				
	≤5 h	6 h	7 h	8 h	≥9 h	≤5 h	6 h	7 h	8 h	≥9 h	≤5 h	6 h	7 h	8 h	≥9 h	≤5 h	6 h	7 h	8 h	≥9 h
**Overall**	14.9	20.4	31.4	24.5	8.8	14.1	20.1	32.5	24.5	8.9	16.1	19.9	30.5	25.6	8.0	16.5	19.5	29.5	25.1	9.4
**Race/ethnicity**																				
White	12.5	19.6	33.7	26.3	7.9	12.0	19.9	33.7	25.5	8.9	14.7	17.7	32.6	27.7	7.3	14.0	19.1	31.5	26.3	9.1
Black	21.0	24.4	26.6	19.5	8.5	22.3	23.4	26.9	19.1	8.3	21.9	26.5	23.7	19.3	8.6	23.7	23.2	24.3	20.0	8.8
Hispanic	15.9	18.4	30.3	23.7	11.7	12.7	17.9	33.3	26.9	9.1	15.5	21.3	29.5	24.8	8.8	17.6	19.1	27.9	25.1	10.3
Other	20.3	24.8	24.4	22.5	8.0	17.9	22.5	29.0	21.1	9.5	18.7	22.7	27.5	21.3	9.8	21.1	17.2	29.2	24.2	8.2
**Grade**																				
9th	12.4	13.6	29.0	31.2	13.8	11.7	14.9	30.6	28.4	14.4	13.1	15.2	28.6	32.1	10.9	13.6	13.8	27.6	30.2	14.8
10th	11.9	19.6	32.9	27.3	8.4	13.0	20.4	33.1	25.2	8.2	13.7	18.2	32.2	26.0	9.9	14.8	18.8	29.3	27.9	9.2
11th	17.9	22.4	32.4	20.4	6.9	15.3	23.1	33.9	21.6	6.1	19.1	22.8	29.5	23.1	5.6	17.2	22.8	30.5	22.6	6.8
12th	18.1	28.4	31.9	16.9	4.7	16.9	23.6	32.4	21.6	5.4	18.8	24.6	31.8	20.2	4.6	20.8	23.7	31.2	18.7	5.6
**White, by grade**																				
9th	11.3	11.7	31.6	33.7	11.7	8.9	14.0	32.1	29.4	15.6	12.2	12.4	30.4	34.8	10.1	9.6	14.2	28.8	33.3	14.2
10th	9.9	17.5	35.1	30.3	7.2	10.9	21.0	34.0	25.9	8.2	12.1	15.6	34.5	28.4	9.3	13.1	17.9	29.9	28.1	10.9
11th	14.7	21.9	34.1	21.9	7.5	13.4	22.9	35.4	23.1	5.2	17.2	21.0	30.4	26.6	4.9	15.4	22.7	33.4	23.4	5.1
12th	14.6	28.8	34.4	17.6	4.6	15.2	22.5	33.4	23.1	5.9	17.2	22.5	35.3	20.5	4.5	18.1	22.2	33.9	20.2	5.5
**Black, by grade**																				
9th	16.5	18.5	22.5	28.2	14.3	20.3	22.8	26.2	19.6	11.1	13.3	22.6	26.0	27.6	10.5	24.2	17.8	22.3	23.1	12.6
10th	16.7	29.1	27.2	18.6	8.4	20.9	19.4	26.6	24.9	8.1	24.7	24.5	22.3	19.1	9.4	22.0	21.6	24.6	25.5	6.4
11th	27.0	24.1	32.7	11.4	4.8	25.9	25.3	29.0	12.3	7.5	23.2	30.4	22.9	15.2	8.4	21.3	26.8	25.8	16.8	9.3
12th	27.7	28.2	26.5	14.5	3.1	23.4	28.1	26.4	17.3	4.7	29.4	31.3	22.8	12.0	4.6	27.2	28.9	25.4	12.9	5.5
**Hispanic, by grade**																				
9th	10.6	14.5	28.4	27.8	18.7	10.8	10.4	32.0	32.8	14.0	15.9	16.2	26.1	30.4	11.4	14.1	12.9	28.2	28.2	16.6
10th	13.3	15.5	34.2	26.1	11.0	11.8	17.0	35.8	26.6	8.7	10.6	19.3	35.8	24.0	10.3	16.6	18.5	26.7	30.6	7.6
11th	20.1	20.7	30.5	21.2	7.5	13.4	23.0	33.2	23.1	7.3	20.1	25.4	28.9	18.3	7.3	16.5	23.1	30.4	20.8	9.2
12th	23.3	26.2	28.1	16.5	5.9	16.1	25.8	32.2	22.3	3.5	16.1	26.5	26.3	26.0	5.2	24.9	23.8	26.7	18.6	6.0
**Other race/ethnicity, by grade**																				
9th	18.1	16.7	22.4	28.6	14.3	18.9	19.9	20.5	27.1	13.6	14.3	19.5	27.9	23.6	14.7	21.4	10.1	28.7	29.2	10.6
10th	18.2	28.8	27.7	18.5	6.7	15.8	26.3	30.8	20.5	6.7	14.4	23.9	24.9	23.2	13.6	13.4	17.2	36.4	26.2	6.8
11th	22.6	26.7	23.4	23.5	3.7	17.9	20.7	30.4	20.6	10.5	24.8	21.9	29.7	18.3	5.2	25.4	16.4	22.8	26.7	8.6
12th	23.4	29.0	24.4	17.5	5.7	19.2	23.7	36.1	15.3	5.8	20.9	26.6	28.1	19.9	4.6	24.0	27.7	29.8	12.5	6.0

a All values are percentages.

For each year, a significantly greater percentage of black females than white females reported obtaining 5 or fewer hours of sleep per school night (Table 1). These differences were also found between white males and black males. For each year, more than 1 in 5 black females and 1 in 5 black males reported obtaining 5 or fewer hours of sleep. At least 15% of Hispanic females and white females and at least 12% of Hispanic males and white males reported obtaining 5 or fewer hours of sleep. In contrast, a significantly higher percentage of black females than white females reported obtaining 9 or more hours of sleep on school nights in 3 of the study years (2007, 2009, and 2013). Nevertheless, only 7.9% to 11.4% of black females reported obtaining 9 or more hours of sleep per school night in any year. Among black males, Hispanic males, and white males (Table 2), we found only one significant difference in the percentage that obtained 9 or more hours of sleep: in 2007, a larger percentage of Hispanic males (11.7%; 95% CI, 9.7%–13.8%) than white males (7.9%; CI, 7.0%–8.7%) reported obtaining 9 or more hours of sleep. Across all 4 waves of data collection, a small (<10%) overall percentage of females and males reported obtaining 9 or more hours of sleep. In 3 years (2007, 2009, and 2013), we found among females (compared with males) both a higher percentage who obtained 5 or fewer hours of sleep and a lower percentage who obtained 9 or more hours of sleep.

## Discussion

The Centers for Disease Control and Prevention recommends that adolescents obtain 9 or 10 hours of sleep each day (8). In our study, a large majority of American high school students did not meet this recommendation. Among 12th graders, approximately 95% of males and females did not meet this recommendation. Data on students that report sleeping 7 hours or fewer on an average school night show that a substantial proportion of American high school students are not even close to obtaining the recommended amount of sleep.

Consistent with prior reports (6), our study shows that the percentage of students obtaining an insufficient amount of sleep increased as students progressed from grades 9 to 12. These findings indicate a need for early intervention, both before and during high school. Although a higher percentage of females than males, especially black females, reported receiving 5 or fewer hours of sleep, the data show a need for universal interventions — those directed toward all students. For example, the American Academy of Pediatrics has encouraged school districts to establish start times that optimize students’ sleep (9). Unfortunately, intervention research directed toward high school populations has received little attention.

Although the important role of sleep in memory was recognized more than a century ago (5), more recent research has clarified the causal mechanisms through which sleep benefits memory, namely by active consolidation of memories through the reactivation of newly encoded memory representations that become incorporated into long-term knowledge (5,10). These insights, coupled with research demonstrating the importance of sleep for emotional self-regulation (6,10), obesity, safety, and attention to on-task learning (6) and the effect of sleep on chronic disease risk factors such as glucose metabolism (11), hypertension (1), inflammatory markers (1,12), and impaired immune response (12), suggest that adequate sleep has implications for academic achievement and the prevention of chronic disease (9). Attention is needed to develop feasible and effective interventions to increase sleep among American high school students.
